# Metabolomic and lipidomic profiling of traditional Chinese medicine *Testudinis Carapax et Plastrum* and its substitutes

**DOI:** 10.3389/fphar.2025.1549834

**Published:** 2025-03-21

**Authors:** Mengru Xin, Yaodong Ping, Yisheng Zhang, Wenqing Zhang, Lin Zhang, Yonghong Zhang, Wentao Sheng, Lei Wang, Weidong Mao, Ling Xiao, Shan Guo, Hankun Hu

**Affiliations:** ^1^ Department of Pharmacy, Zhongnan Hospital of Wuhan University, School of Pharmaceutical Sciences, Wuhan University, Wuhan, China; ^2^ Key laboratory of Carcinogenesis and Translational Research (Ministry of Education/Beijing), Department of Pharmacy, Peking University Cancer Hospital and Institute, Beijing, China; ^3^ Department of Pharmacy, Wuhan Hospital of Traditional Chinese Medicine, Wuhan, Hubei, China; ^4^ Hubei Shizhen Laboratory, School of Basic Medical Sciences, Hubei University of Chinese Medicine, Wuhan, China; ^5^ Laboratory of Medicinal Plant, Hubei Key Laboratory of Embryonic Stem Cell Research, Academy of Bio-Medicine Research, School of Basic Medicine, Hubei University of Medicine, Shiyan, China; ^6^ Hubei Shengchang Aquatic Products Co., Ltd., Jingshan, Hubei, China; ^7^ Hubei Laozhongyi Pharmaceutical Co., Ltd., Xiaogan, Hubei, China; ^8^ Department of Information Technology, Georgia Gwinnett College, Lawrenceville, GA, United States; ^9^ Hubei Institute for Drug Control, NMPA Key Laboratory of Quality Control of Chinese Medicine Hubei, Engineering Research Center for Drug Quality Control, Wuhan, China; ^10^ Department of Biological Repositories, Zhongnan Hospital of Wuhan University, Wuhan, Hubei, China

**Keywords:** *Testudinis carapax et Plastrum*, liquid chromatography-tandem mass spectrometry, metabolomic and lipidomic analyses, metabolites, lipids

## Abstract

**Introduction:**

*Chinemys reevesii* (Gray) species–sourced *Testudinis Carapax et Plastrum* (TCP) is an animal-based traditional Chinese medical material, and its decoction or extract possesses multiple pharmacological effects. However, other species-sourced substitutes are sometimes used in the market, potentially impairing the quality and effectiveness of TCP medications. To address this issue, it is very necessary to develop applicable approaches that can accurately differentiate genuine TCP from its counterfeit counterparts.

**Methods:**

In this study, liquid chromatography–tandem mass spectrometry (LC-MS/MS)–based metabolomic and lipidomic analyses were performed to comprehensively detect water-soluble metabolites and organic-soluble lipids in water decoctions of genuine TCP and its substitutes, such as *Trachemys scripta elegans* (Wied)– and *Ocadia sinensis* (Gray)–sourced tortoise shells. Differential analyses based on fold change (FC), principal component analysis (PCA), and Orthogonal partial least squares–discriminant analysis (OPLS-DA) were performed to assess the differences among TCP decoctions from different origins, as well as between decoctions of TCP samples and the two substitutes. Further, Kyoto Encyclopedia of Genes and Genomes (KEGG) database–based pathway enrichment analysis was performed for differential metabolites and lipids among them. Besides, LC-MS/MS–based absolute quantitative method was used to quantify the amino acid–relevant metabolites in decoctions of TCP and substituted tortoise shell samples.

**Results:**

All told, 1117 water-soluble metabolites (including amino acids, organic acids, nucleotides and their metabolites or derivatives, etc.) and 574 organic-soluble lipids (including glycerolipids, sphingolipids, glycerophospholipids, fatty acids, and sterol lipids) were detected in decoctions of TCP and two substitutes. Comparative analyses revealed that there were significantly differential metabolites and lipids among TCP decoctions from different origins, as well as between decoctions of TCP samples and the two substitutes. Of particular interest, the content of N-methyl-4-aminobutyric acid was lower in the substituted samples than TCP samples. Furthermore, the content of 27 amino acids, 22 amino acid derivatives, and 18 small peptides in the decoctions of TCP and two substitutes were absolutely quantified, constituting up to tens of milligrams per 10 g of tortoise shell.

**Discussion:**

In conclusion, our study provides comprehensive metabolomic and lipidomic information of TCP decoction. However, the current results represent preliminary data, and further extensive research is required to validate these findings.

## 1 Introduction

*Testudinis Carapax et Plastrum* (TCP), often referred to as “guijia” in Chinese, is the dried carapace and plastron from *Chinemys reevesii* (syn. *Mauremys reevesii Gray*, 1831) (Commission, 2020). The *Pharmacopoeia of the People’s Republic of China* (2020 edition) lists that TCP is salty, sweet in taste and slightly in nature; and passes through the liver, kidney, and heart channels. TCP’s main indications include nourishing yin and latent yang, invigorating the kidneys and strengthening bone, nourishing blood and tonifying the heart, and reinforcing the meridian and stopping collapse. Current studies have demonstrated that the extracts of *Plastrum Testudinis* (i.e., the dried plastron of *Chinemys reevesii*) have protective effects against bone diseases (e.g., senile osteoporosis, intervertebral disc degeneration) (Liang et al., 2016; Ren et al., 2017; Chen et al., 2021; Zhang et al., 2021; Zhang et al., 2023), acute promyelocytic leukemia (Chen et al., 2024), Parkinson’s disease (Zhong et al., 2020; Ye et al., 2021; Yi et al., 2024), beta-thalassemia and sickle cell anemia (Qian et al., 2013), Alzheimer’s disease (Hui et al., 2017), and skin wounds (Tang et al., 2018).

TCP materials are typically ground into a powder and then decocted in water for therapeutic use. TCP medications can be taken internally or externally. Water decoctions of TCP can be further made into *Testudinis Carapacis et Plastri Colla* (named as “guijia jiao” in Chinese) (Su et al., 2018; Commission, 2020). Various TCP-associated Chinese patent drugs are available on the market, such as Gulu Erxian Gao, Yangyin Jiangya Jiaonang, and Jianbu Qiangshen Wan. These processed TCP products have lost their original morphological characteristics (including shape, color, texture, etc.), thereby creating critical vulnerabilities for economically motivated adulteration within pharmaceutical supply chains. For instance, TCP powders are sometimes mixed with low-cost substitutes from other species-sourced tortoise shells, such as carapace- or plastron-derived materials from *Ocadia sinensis* (Gray) and *Trachemys scripta elegans* (Wied). Such adulteration may compromise the bioactive composition of TCP decoctions, ultimately diminishing their therapeutic efficacy. It is therefore urgent to develop applicable approaches allowing accurate distinguishment of TCP samples from counterfeits.

Currently, polymerase chain reaction (PCR)-based DNA molecular strategies, such as quantitative PCR and multiplex PCR, have been leveraged to identify species of tortoise shells (Jiao et al., 2020; Lv et al., 2020). Species-specific primers have been designed for genomic DNA and mitochondrial DNA of TCP (Li et al., 2018; Yang et al., 2018). However, such approaches are limited to assessing highly processed TCP samples with trace amounts of degraded DNA. Metabolites that are small molecules (typically <2,000 Da) are chemically transformed during metabolism, reflecting the direct signature of biochemical activity in the organism (Sarmad et al., 2023). The chemical constituents detected in TCP extracts include fatty acids, steroids, amino acids, peptides, etc. (Wang et al., 2012; Ren et al., 2024). Thus, system-wide profiling of metabolites, including lipids, in TCP samples and counterfeits may reveal the characteristic metabolites that may be used as novel indicators to discriminate adulteration in processed TCP samples. With advances in mass spectrometry (MS)-based metabolomics and lipidomic analyses, thousands of metabolites and lipids that span a large dynamic range can now be quantitatively measured in a single sample (Guo et al., 2020; Alseekh et al., 2021; Perez de Souza et al., 2021). Gas chromatograph–MS (GC-MS)– and liquid chromatography–MS (LC-MS)–based metabolomic and lipidomic analyses have been performed to authenticate traditional Chinese medicine materials, such as cordyceps (Zhang et al., 2020; Lin et al., 2022; Hou et al., 2025). In comparison to GC-MS, which is primarily limited to volatile and thermally stable compounds, LC-MS demonstrates superior analytical capability for a wider spectrum of analytes, particularly non-volatile and thermally labile compounds.

In this study, we used LC-MS/MS–based widely targeted metabolomic and lipidomic approaches to comprehensively analyze both water-soluble metabolites and organic-soluble lipids in decoctions of *Chinemys reevesii* (Gray) species–sourced TCP and other species-sourced substitutes. Univariate and multivariate statistical analyses were carried out to assess differences in decoctions of TCP samples collected from different places and those in decoctions of TCP samples and two substitutes, including *Trachemys scripta elegans* (Wied)– and *Ocadia sinensis* (Gray)–sourced tortoise shells collected from different places. Furthermore, targeted quantification was conducted to evaluate the contents of amino acids and their metabolites across specimen groups.

## 2 Materials and methods

### 2.1 Tortoise shell samples and their decoction preparation

*Chinemys reevesii* (Gray) species–sourced TCP samples (including CR1, CR2, and CR3), along with *Trachemys scripta elegans* (Wied)– and *Ocadia sinensis* (Gray)–sourced tortoise shells (including TS1, TS2, OS1, and OS2), were provided by the Hubei Institute for Drug Control (Wuhan, China) (Table 1). The authentication of CR, TS, and OS samples was performed by Professor Ling Xiao, Chief Pharmacist at the Hubei Institute for Drug Control (Wuhan, China). CR1, OS1, and TS1 samples were sourced from Jingshan, Hubei, China. CR2, OS2, and TS2 samples were collected from Hanshou, Hunan, China. CR3 samples were collected from Jingzhou, Hubei, China.

**TABLE 1:** Information of genuine and counterfeit TCP samples.

Tortoise shell samples	Species	Collection site	Identification
CR1	*Chinemys reevesii* (Gray)	Jingshan, Hubei, China	Genuine TCP
CR2	*Chinemys reevesii* (Gray)	Hanshou, Hunan, China	Genuine TCP
CR3	*Chinemys reevesii* (Gray)	Jingzhou, Hubei, China	Genuine TCP
OS1	*Ocadia sinensis* (Gray)	Jingshan, Hubei, China	Counterfeit TCP
OS2	*Ocadia sinensis* (Gray)	Hanshou, Hunan, China	Counterfeit TCP
TS1	*Trachemys scripta elegans* (Wied)	Jingshan, Hubei, China	Counterfeit TCP
TS2	*Trachemys scripta elegans* (Wied)	Hanshou, Hunan, China	Counterfeit TCP

To prepare decoctions of CR, TS, and OS samples, 100 mL of distilled water was added to 10 g of each sample, which was subsequently soaked for 1 h. Then, the mixture was heated under reflux conditions and kept boiling slightly for 8 h. Finally, the mixture was cooled to room temperature and filtered. The filtered solution was stored at −80°C for later use.

### 2.2 Chemical Reagents

Acetonitrile, methanol, isopropanol, and methyl-tert-butyl ether (MTBE) were purchased from Merck (Darmstadt, Germany). Formic acid, ammonium hydroxide, and ammonium acetate were purchased from Sigma-Aldrich (St. Louis, MO, United States), while ammonium formate was purchased from Thermo Fisher Scientific (Waltham, MA, United States). Ultrapure water was obtained using a Milli-Q system (Millipore, Billerica, MA, United States). Standards for quantitative analysis of amino acid–relevant metabolites are shown in Supplementary Table S1.

### 2.3 LC-MS/MS–based metabolomic and lipidomic analyses of tortoise shell decoctions

#### 2.3.1 Analysis of water-soluble metabolites in decoctions

To extract water-soluble metabolites, 150 μL of acetonitrile/methanol (1:4, v/v) was added to 50 μL of each decoction sample and vortexed for 3 min (Want et al., 2006). Then, the mixture was centrifuged at 12,000 rpm for 10 min at 4°C, and 150 μL of supernatant was collected. To precipitate the proteins in the samples as far as possible, the supernatant was stored at −20°C for 30 min. Finally, the supernatant was centrifuged again at 12,000 rpm for 3 min at 4°C, and 120 μL of supernatant was collected for LC-MS/MS analysis.

For LC separation, an ExionLC AD UPLC system (SCIEX, Framingham, MA, United States) was used to separate water-soluble metabolites. The injection volume was 2 μL. A Waters ACQUITY UPLC HSS T3 C18 column (2.1 mm × 100 mm, 1.8 µm) was used and its temperature was maintained at 40°C. The mobile phases were composed of (A) water with 0.1% formic acid and (B) acetonitrile with 0.1% formic acid. The flow rate was 0.4 mL/min. The gradient elution was performed as follows: 0–11 min, 5%–90% B; 11–12 min, 90% B; 12–12.1 min, 90%–5% B; and 12.1–14 min, 5% B.

MS data acquired in the multiple reaction monitoring (MRM) mode were performed on a QTRAP^®^ 6500+ MS system (SCIEX, Framingham, MA, United States) equipped with an electrospray ionization source. Parameters were set as follows: source temperature 500°C; positive ion spray voltage, 5,500 V; negative ion spray voltage, −4,500 V; GSI, 55 psi; GSII, 60 psi; curtain gas, 25 psi; and collision gas, high.

#### 2.3.2 Analysis of organic-soluble lipids in decoctions

To extract organic-soluble lipids, 1 mL of MTBE/methanol (3:1, v/v) was added to 200 μL of each decoction sample, which was vortexed for 15 min (Matyash et al., 2008). Then, 100 μL of water was added to the mixture, which was followed by vortexing for 1 min and then centrifugation at 12,000 rpm for 10 min at 4°C to achieve phase separation. Subsequently, 500 μL of upper organic phase was collected, concentrated, and reconstituted in 200 μL of acetonitrile/isopropanol (1:1, v/v). Finally, the reconstituted sample was centrifuged at 12,000 rpm for 3 min at 4°C, and the supernatant was collected for LC-MS/MS analysis.

For LC separation, an ExionLC AD UPLC system (SCIEX, Framingham, MA, United States) was used to separate lipids. A Thermo Accucore™ C30 column (2.1 mm × 100 mm, 2.6 µm) was used, and its temperature was maintained at 45°C. The injection volume was 2 μL. The mobile phases were composed of (A) acetonitrile/water (60:40, v/v) with 0.1% formic acid and 10 mmol/L of ammonium formate and (B) acetonitrile/isopropanol (10:90, v/v) with 0.1% formic acid and 10 mmol/L of ammonium formate. The flow rate was 0.35 mL/min. The gradient elution was performed as follows: 0–2 min, 20%–30% B; 2–4 min, 30%–60% B; 4–9 min, 60%–85% B; 9–14 min, 85%–90% B; 14–15.5 min, 90%–95% B; 15.5–17.3 min, 95% B; 17.3–17.5 min, 95%–20% B; and 17.5–20 min, 20% B.

MS data acquired in MRM mode were also performed on a QTRAP^®^ 6500+ MS system (SCIEX, Framingham, MA, United States). Parameters were set as follows: source temperature 500°C; positive ion spray voltage, 5,500 V; negative ion spray voltage, −4,500 V; GSI, 45 psi; GSII, 55 psi; and curtain gas, 35 psi; collision gas, medium.

#### 2.3.3 Data processing

Water-soluble metabolites and organic-soluble lipids were identified in Analyst 1.6.3 (SCIEX, Framingham, MA, United States) based on retention time, precursor ion/product ion information, and MS/MS spectrum patterns from a self-compiled database (Metware Biotechnology Co., Ltd., Wuhan, China) and public databases including the Metabolite Link database (METLIN; https://massconsortium.com/) and Human Metabolome Database (HMDB; https://hmdb.ca/). Integration and calibration of the chromatographic peaks were performed in MultiQuant 3.0.3 (SCIEX, Framingham, MA, United States). Data of water-soluble metabolites and organic-soluble lipids detected in quality control (QC) samples with a peak area coefficient of variation of ≥30% were filtered out and not analyzed. QC samples were prepared by mixing small aliquots of each decoction from tortoise shells.

Principal component analysis (PCA) was performed in R (base package, version 4.1.2; R Foundation for Statistical Computing, Vienna, Austria). Orthogonal partial least squares–discriminant analysis (OPLS-DA) was performed by using the R package MetaboAnalystR (version 1.0.1). Differential metabolites and lipids were screened based on the combination of a statistically significant threshold of variable importance in projection (VIP) ≥ 1 and |Log_2_FC| ≥ 1.0.

The identified water-soluble metabolites and organic-soluble lipids in decoctions were annotated using the Kyoto Encyclopedia of Genes and Genomes (KEGG) Compound database (http://www.kegg.jp/kegg/compound/). Annotated metabolites and lipids were then mapped to the KEGG Pathway database (http://www.kegg.jp/kegg/pathway.html). Pathways containing significantly regulated metabolites and lipids were subsequently subjected to metabolite set enrichment analysis.

### 2.4 LC-MS/MS–based quantitative analysis of amino acid–relevant metabolites in decoctions

Here, 250 μL of acetonitrile/methanol (1:4, v/v) was added to 50 μL of each decoction sample, followed by vortexing for 3 min. Then, the mixture was centrifuged at 12,000 rpm for 10 min at 4°C. In addition, 250 μL of supernatant was collected and kept at −20°C for 30 min to further precipitate the proteins. Finally, the supernatant was centrifuged at 12,000 rpm for 10 min at 4°C, and 180 μL of supernatant was collected for LC-MS/MS–based absolute quantitative analysis. The external standard method was used to quantify 27 amino acids, 22 amino acid derivatives, and 18 small peptides in decoctions of each tortoise shell sample.

LC separation was performed on an ExionLC AD UPLC system (SCIEX, Framingham, MA, United States) with two types of columns, including a Waters ACQUITY HSS T3 C18 column (100 mm × 2.1 mm, 1.8 µm) and a Waters ACQUITY UPLC BEH Amide column (100 mm × 2.1 mm, 1.7 µm) (Supplementary Table S1). The injection volume was 2 μL. The column temperature was maintained at 40°C. For C18 column–based LC separation, the flow rate was 0.35 mL/min. Mobile phases included (A) water with 0.05% formic acid and (B) acetonitrile with 0.05% formic acid. The gradient elution was performed as follows: 0–8 min, 5%–95% B; 8–9.5 min, 95% B; 9.5–9.6 min, 95%–5% B; and 9.6–12 min, 5% B. For amide column–based LC separation, the flow rate was 0.4 mL/min. Mobile phases included (A) water with 0.3% ammonium hydroxide and 10 mM of ammonium acetate and (B) acetonitrile/water (90:10, v/v). The gradient elution was performed as follows: 0–8 min, 5%–95% B; 8–9.5 min, 95% B; 9.5–9.6 min, 95%–5% B; and 9.6–12 min, 5% B.

MS data acquired in MRM mode were performed on a QTRAP^®^ 6500+ LC-MS/MS system (SCIEX, Framingham, MA, United States). Parameters were set as follows: ESI source temperature, 550°C; positive ion spray voltage, 5,500 V; negative ion spray voltage, −4,500 V; GSI, 50 psi; GSII, 60 psi; curtain gas, 35 psi; and collision gas, medium.

## 3 Results

### 3.1 Metabolomic and lipidomic profiling of water-soluble metabolites and organic-soluble lipids in decoctions of genuine TCP and two substitutes

In this work, water-soluble metabolites and organic-soluble lipids in decoctions of *Chinemys reevesii *(Gray) species–sourced tortoise shells (i.e., TCP) and two common substitutes (i.e., *Ocadia sinensis* (Gray) species–sourced and *Trachemys scripta elegans* (Wied) species–sourced tortoise shells) were analyzed by LC-MS/MS–based widely-targeted metabolomic and lipidomic profiling (Table 1). These three species belong to the *Emydidae* family, Testudinate order, Reptilia class (http://museum.ioz.ac.cn/). Genuine TCP samples included CR1, CR2, and CR3. *Ocadia sinensis* (Gray) species–sourced tortoise shell samples included OS1 and OS2. *Trachemys scripta elegans* (Wied) species–sourced tortoise shell samples included TS1 and TS2 (Table 1).

**FIGURE 1:**
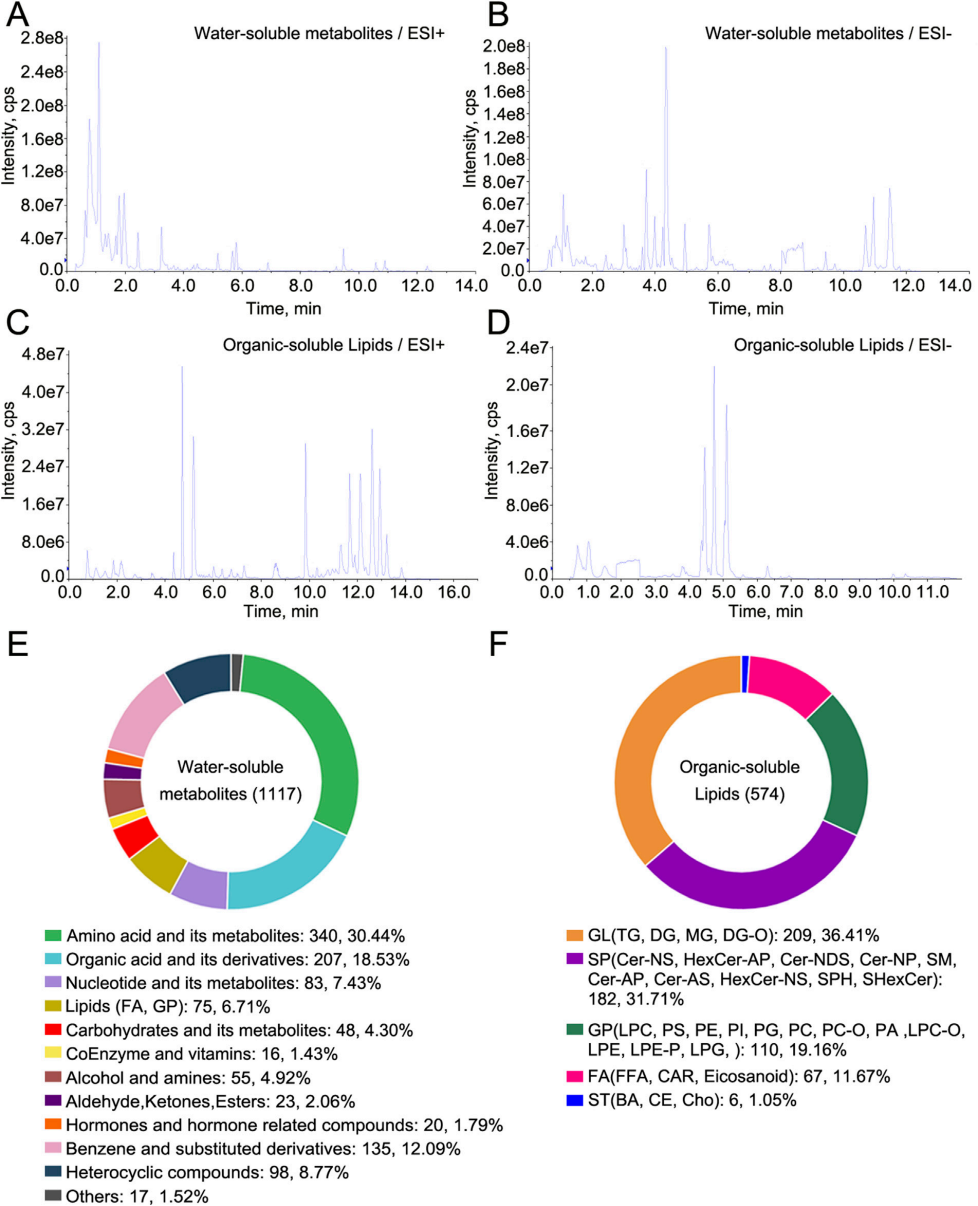
Metabolomic and lipidomic profiling of water-soluble metabolites and organic-soluble lipids in decoctions of TCP and substituted tortoise shells. **(A–D)** Total ion chromatograms of metabolomic and lipidomic analyses of water-soluble metabolites and organic-soluble lipids in ESI+ and ESI− modes. **(E)** Water-soluble metabolites detected in decoctions of tortoise shell samples. **(F)** Organic-soluble lipids detected in decoctions of tortoise shell samples.

Total ion chromatograms of metabolomic and lipidomic analyses of water-soluble metabolites and organic-soluble lipids in the QC sample in ESI+ and ESI− modes are shown in Figure 1A–D. A total of 1,117 water-soluble metabolites and 574 organic-soluble lipids were measured in decoctions of tortoise shell samples, including CR1–3, OS1–2, and TS1–2 (Figures 1E, F; Supplementary Tables S2, S3). No unique metabolites or lipids were found in decoctions of genuine or substituted tortoise shells. The classes of detected water-soluble metabolites consisted of amino acids and their metabolites, organic acids and their derivatives, and nucleotides and their metabolites (Figure 1E). The classes of detected organic-soluble lipids included glycerolipids (GLs), sphingolipids (SPs), glycerophospholipids (GPs), fatty acids (FAs), and sterol lipids (STs) (Figure 1F). Among them, the number of lipids, amino acids, organic acids, and their metabolites or derivatives detected in decoctions was more than 200; in particular, the number of lipids was the highest (649) (Figures 1E, F). These results show that decoction samples of both genuine and substituted tortoise shells contain multiple chemical constituents, providing chemical evidence for parsing functions of tortoise shells.

### 3.2 Differential analyses of metabolites and lipids detected in decoctions of genuine TCP samples from different origins

**FIGURE 2:**
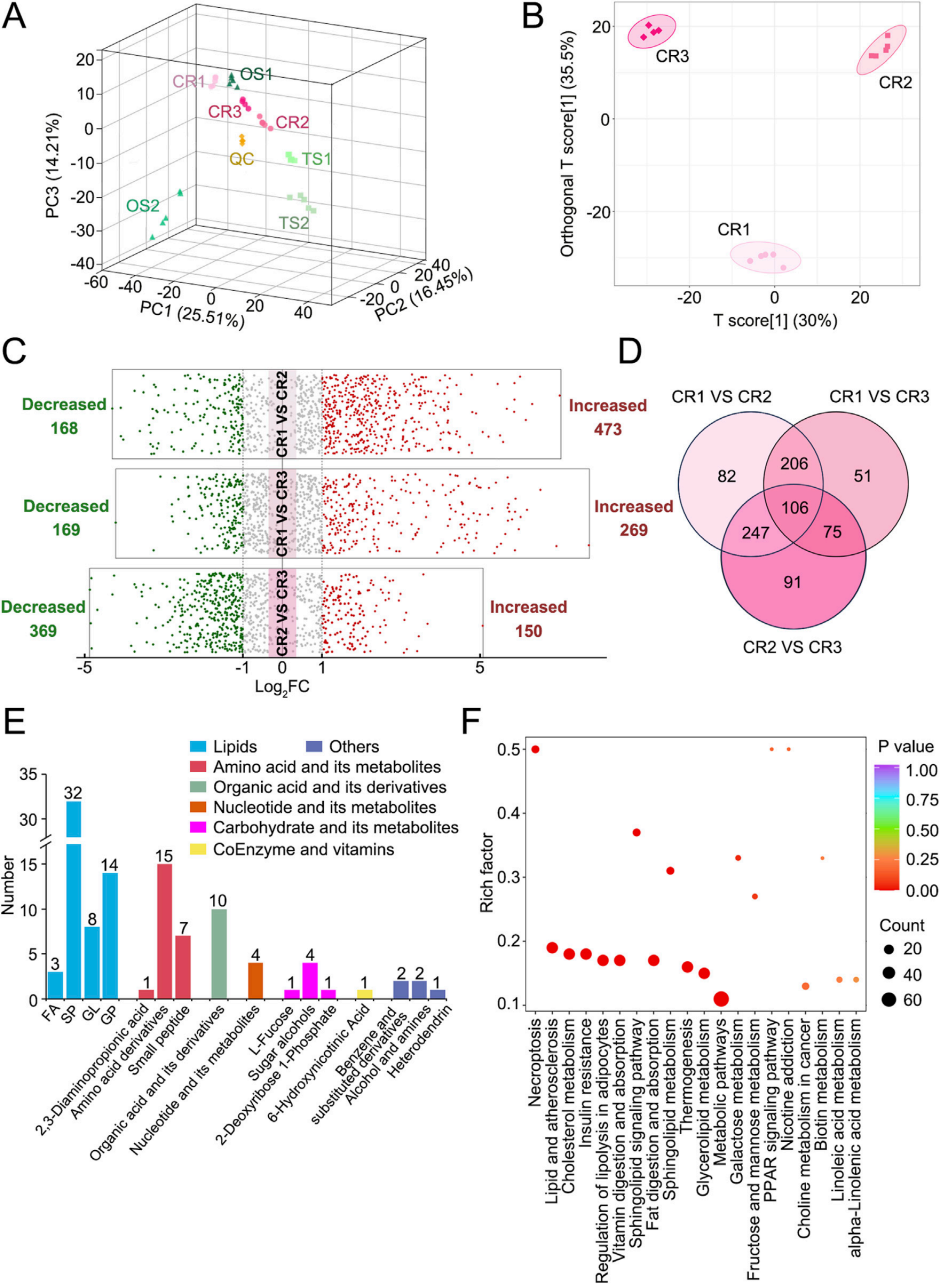
Differential analyses of metabolites and lipids detected in decoctions of genuine TCP samples from different origins, including CR1–3 samples. **(A)** PCA results of CR1–3, OS1–2, and TS1–2 samples. **(B)** OPLS-DA score plot of CR1–3 samples. **(C)** Volcano plots of differential analyses among CR1–3 samples. The filter criteria were |Log_2_FC| ≥ 1.0 and VIP ≥1. **(D)** Venn diagram of differential metabolites and lipids among CR1–3 samples. **(E)** Diagram of common differential metabolites and lipids found among CR1–3 samples. **(F)** Pathway enrichment analysis of common differential metabolites and lipids found among CR1–3 samples. The top 20 enriched pathways as ranked by *p*-value are displayed.

Multivariate statistical analyses, such as PCA and OPLS-DA, clearly showed that CR1–3 sample clusters were separate, suggesting that different water-soluble metabolites and organic-soluble lipids exist in decoctions of genuine TCP samples from different origins (Figures 2A, B). Based on |Log_2_FC| ≥ 1.0 and VIP ≥1, more than 400 differential metabolites and lipids were screened out between CR1 and CR2, between CR1 and CR3, and between CR2 and CR3, respectively (Figure 2C; Supplementary Tables S4–S6). Among them, the number of common differential metabolites and lipids was 106 (Figure 2D). The classes of common differential substances included lipids, amino acids and their metabolites, organic acids and their derivatives, nucleotides and their metabolites, carbohydrates and their metabolites, coenzymes, and vitamins (Figure 2E), which are highlighted in bold in Supplementary Tables S4–S6. More common differential metabolites and lipids were significantly enriched into metabolic pathway and lipid metabolism–related pathways, such as those relating to cholesterol metabolism, glycerolipid metabolism, sphingolipid signaling, fat digestion and absorption, and regulation of lipolysis in adipocytes (Figure 2F). Taken together, there were significant differences in decoctions of genuine TCP samples from different origins.

### 3.3 Differential analyses of metabolites and lipids detected in decoctions of genuine TCP samples and *Ocadia sinensis* (Gray) species–sourced substituted tortoise shell samples

**FIGURE 3:**
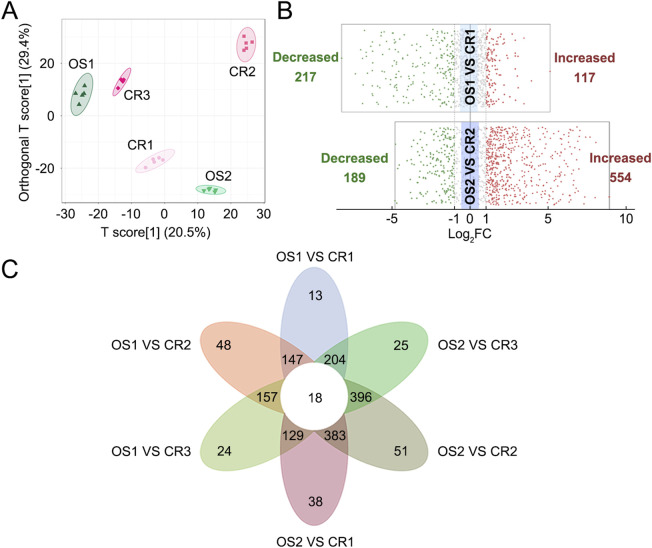
Differential analyses of metabolites and lipids detected in decoctions of CR samples and OS samples. **(A)** OPLS-DA score plot of CR1–3 and OS1–2 samples. **(B)** Volcano plots of differential analyses between CR and OS samples from the same origins. The filter criteria were |Log_2_FC| ≥ 1.0 and VIP ≥1. **(C)** Venn diagram of differential metabolites and lipids found between CR1–3 and OS1–2 samples.

PCA and OPLS-DA results revealed five distinct sample clusters for CR1, CR2, CR3, OS1, and OS2 (Figure 3A), indicating that decoctions of substituted tortoise shell samples OS1–2 from different origins differ from those of genuine TCP samples CR1–3. Based on |Log_2_FC| ≥ 1.0 and VIP ≥1, 334 and 743 differential metabolites and lipids were sifted out between CR1 and OS1 and between CR2 and OS2, respectively (Figure 3B; Supplementary Tables S7, S8). Besides, decoctions of genuine TCP and substituted OS tortoise shell samples from different origins also demonstrated different metabolites and lipids (Figure 3C). In all, 18 common differential metabolites and lipids were screened out between CR and OS samples, including 13 lipids (i.e., 2 FAs, 1 DG, 4 TGs, 4 LPCs, and 2 Cers), two amino acids and their metabolites (N-lactoyl-phenylalanine and N-methyl-4–aminobutyric acid), one organic acid and its derivatives (3-methylcrotonyl glycine), one benzene and substituted derivatives (3-sulfocatechol), and one heterocyclic compound (imidazoleacetic acid) (Figure 3C; Table 2), which are highlighted in bold in Supplementary Tables S7, S8. KEGG pathway enrichment analysis showed that 18 differential metabolites and lipids were significantly enriched in cell growth and death, lipid metabolism, and endocrine and metabolic disease pathways (*p* < 0.05) (Supplementary Figure S1A). These results indicate that metabolic and lipidomic profiling of decoctions of genuine TCP samples from different origins are different from those of substituted OS tortoise shell samples.

**TABLE 2:** Common differential metabolites and lipids found between CR and OS samples.

Number	Class	Subclass	Metabolites and lipids
1	Lipids	FA	FFA (20:5)
2			(±)5-HEPE
3		GL	DG (O-19:2_20:0)
4			TG (14:0_18:0_20:2)
5			TG (16:0_18:0_20:1)
6			TG (16:0_18:1_22:1)
7			TG (18:1_18:1_22:1)
8		GP	LPC(O-14:0)
9			LPC(O-18:0)
10			LPC(O-18:1)
11			LPC(O-18:2)
12		SP	Cer(d16:0/18:0)
13			Cer(t17:2/22:0)
14	Amino acids and metabolites	Amino acid derivatives	N-lactoyl-phenylalanine
15			N-methyl-4–aminobutyric acid
16	Organic acids and derivatives	Organic acids and derivatives	3-Methylcrotonyl glycine
17	Others	Benzene and substituted derivatives	3-Sulfocatechol
18		Heterocyclic compounds	Imidazoleacetic acid

### 3.4 Differential analyses of metabolites and lipids detected in decoctions of genuine TCP and *Trachemys scripta elegans* (Wied)–sourced substituted tortoise shell samples

**FIGURE 4:**
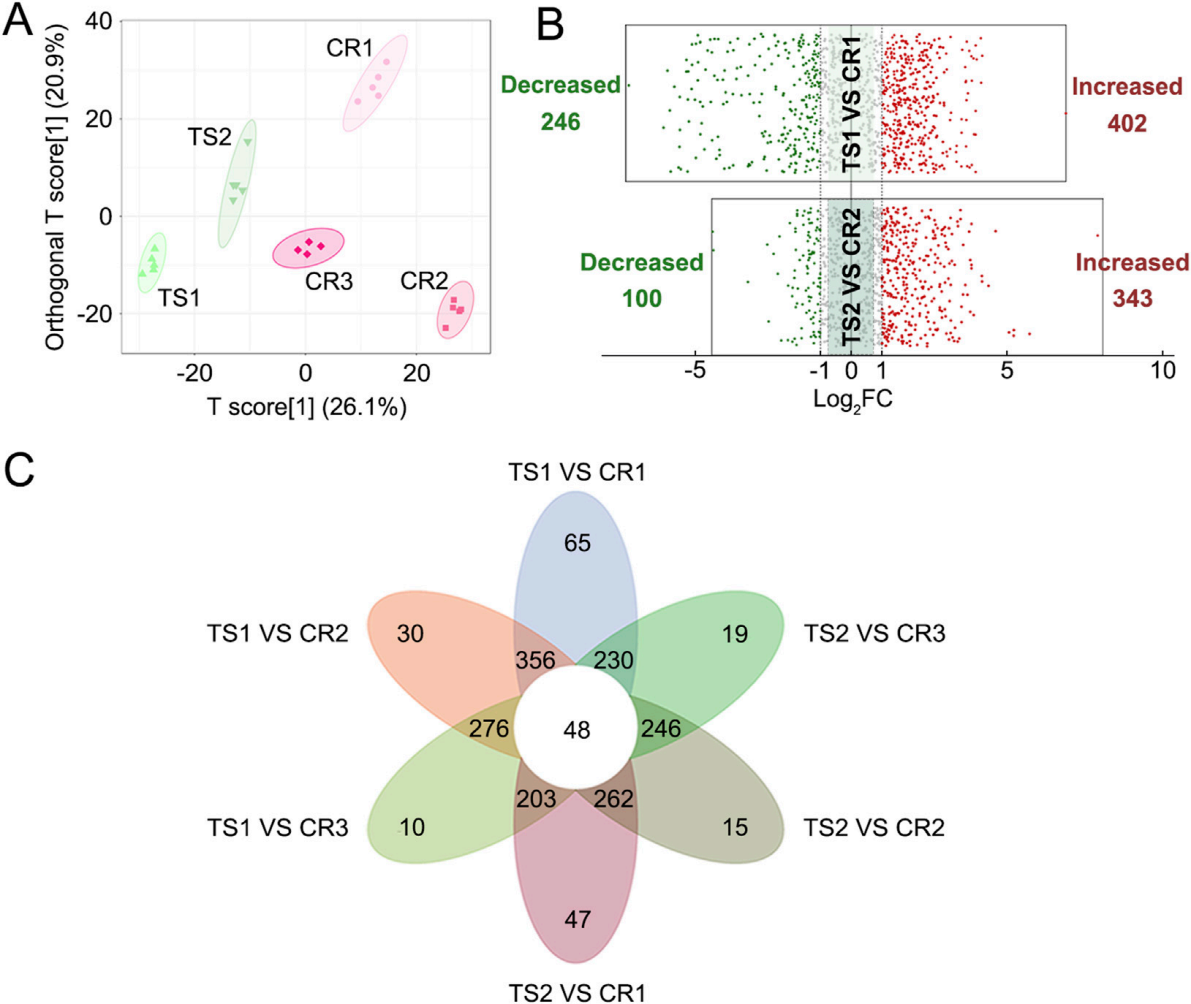
Differential analyses of metabolites and lipids detected in decoctions of CR1–3 samples and TS1–2 samples. **(A)** OPLS-DA score plot of CR1–3 and TS1–2 samples. **(B)** Volcano plots of differential analyses between CR and TS samples from the same origins. The filter criteria were |Log_2_FC| ≥ 1.0 and VIP ≥1. **(C)** Venn diagram of differential metabolites and lipids found between CR1–3 and TS1–2 samples.

**FIGURE 5:**
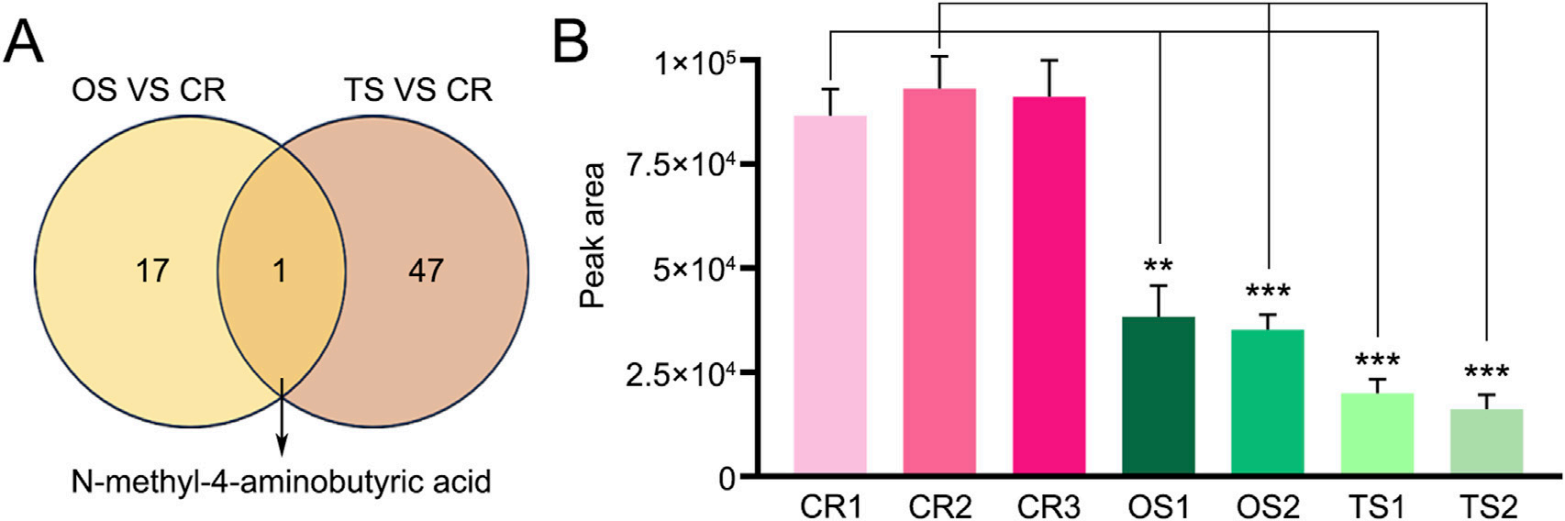
Display of the only common differential (N-methyl-4–aminobutyric acid) found in decoctions of genuine TCP samples and substituted OS and TS samples. **(A)** Venn diagram of differential metabolites or lipids found between CR, OS, and TS samples. **(B)** Levels of N-methyl-4–aminobutyric acid in decoctions of CR, OS, and TS samples. Data are presented as mean ± SD, n = 5. **: *p* < 0.01; ***: *p* < 0.001.

PCA and OPLS-DA results also showed significant differences between TCP samples (CR1–3) and substituted tortoise shell samples (TS1–2) (Figures 2A, 4A). Based on |Log_2_FC| ≥ 1.0 and VIP ≥1, more than 400 differential metabolites and lipids were found between CR and TS samples from the same origins (Figure 4B; Supplementary Tables S9, S10). All told, 48 common differential metabolites and lipids were found between CR1–3 and TS1–2 samples, including 12 lipids, 11 amino acids and their metabolites, 6 organic acids and their derivatives, 12 nucleotides and their metabolites, 1 carbohydrate and its metabolites (2,4-diacetamino-2,4,6-triphenoxy-D-mannopyranose), 1 coenzyme or vitamin (riboflavin), and 5 other compounds (putrescine, carbendazim, ectoine, N′-methyl-2-pyridone-5-carboxamide, and furfural diacetal) (Figure 4C; Table 3), which are highlighted in bold in Supplementary Tables S9, S10. KEGG pathway enrichment analysis revealed that 48 differential metabolites and lipids were significantly enriched in multiple metabolic pathways, such as those associated with nicotinate and nicotinamide metabolism, purine metabolism, nucleotide metabolism, sphingolipid metabolism, and butanoate metabolism (*p* < 0.05) (Supplementary Figure S1B). Furthermore, it was found that the N-methyl-4–aminobutyric acid content was significantly lower in decoctions of substituted tortoise shell samples (OS1–2 and TS1–2) compared to in genuine TCP samples (*p* < 0.01) (Figures 5A, B). These results further suggest that metabolic and lipidomic profiling of decoctions of genuine TCP samples from different origins differ from that of substituted TS tortoise shell samples.

**TABLE 3:** Common differential metabolites and lipids found between CR and TS samples.

Number	Class	Subclass	Metabolites and lipids
1	Lipids	FA	PGF2α
2			12-Hydroxyoctadecanoic acid
3		SP	Cer(t18:0/24:0(2OH))
4			Cer(t20:0/24:0(2OH))
5			Cer(t18:0/24:1)
6			Cer(d16:1/24:2)
7			Cer(d20:1/28:2)
8			HexCer(t22:2/14:1(2OH))
9			HexCer(d18:1/18:0)
10			HexCer(d18:1/18:1)
11			HexCer(d18:1/20:0)
12			HexCer(d18:1/20:1)
13	Amino acids and metabolites	Amino acid derivatives	2–Aminoisobutyric acid
14			Aminoisobutyric acid
15			N, N-dimethylglycine
16			N-methyl-4–aminobutyric acid
17		Small peptides	Ala-Tyr
18			Arg-Ile
19			Glu-Phe
20			Leu-Asp
21			Met-Phe
22			Phe-Glu
23			Tyr-Ser
24	Organic acids and derivatives	Sulfonic acids	p-Tolyl sulfate
25		Carboxylic acids and derivatives	Cinnamic acid
26			Fumaric acid
27			L-2–aminobutyric acid
28			Maleic acid
29			Shikimic acid
30	Nucleotides and metabolites	Purines and purine derivatives	1-Methyladenosine
31			2-Aminopurine
32		Purine nucleosides	2′-Deoxyadenosine
33			2′-Deoxyinosine
34			3-Deoxyadenosine
35			5′-Deoxy-5′-fluoroadenosine
36			5′-Deoxyadenosine
37			Adenosine
38			Arainosine
39			Guanosine
40			N6-methyl-2′-deoxyadenosine
41			N6-methyladenosine
42	Carbohydrates and metabolites	Sugar derivatives	2,4-diacetamino-2,4,6-triphenoxy-D-mannopyranose
43	Coenzymes and vitamins	Coenzymes and vitamins	Riboflavin
44	Others	Alcohols and amines	Putrescine
45		Heterocyclic compounds	Carbendazim
46			Ectoine
47			N'-methyl-2-pyridone-5-carboxamide
48		Others	Furfural diacetal

### 3.5 Quantitative analyses of amino acids and their metabolites in decoctions of genuine and substituted tortoise shell samples

**FIGURE 6:**
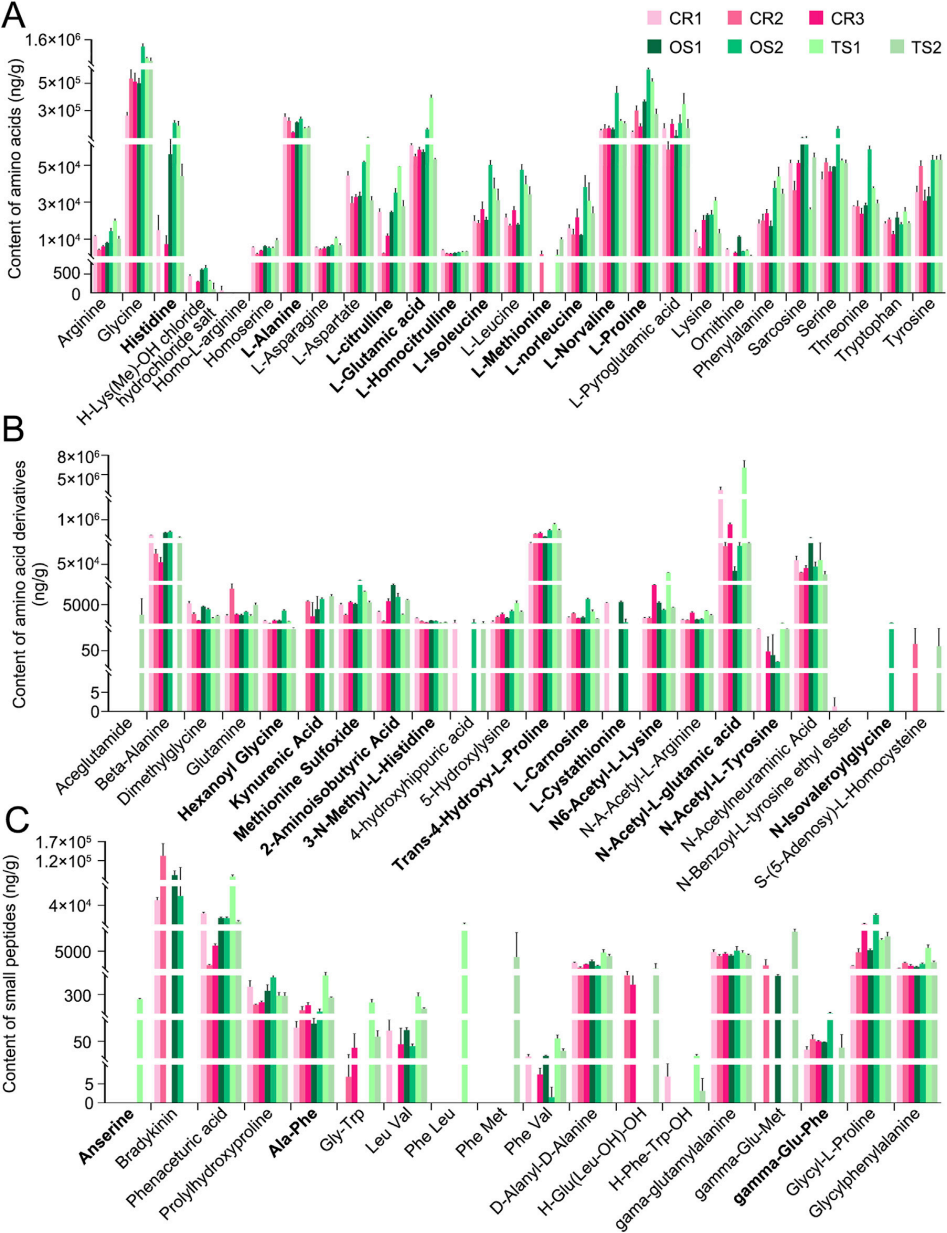
Absolute quantitative analysis results of some amino acids and their metabolites in decoctions of CR, OS, and TS samples. **(A)** Amino acids. **(B)** Amino acid derivatives. **(C)** Small peptides. Data are presented as mean ± SD, n = 3.

**FIGURE 7:**
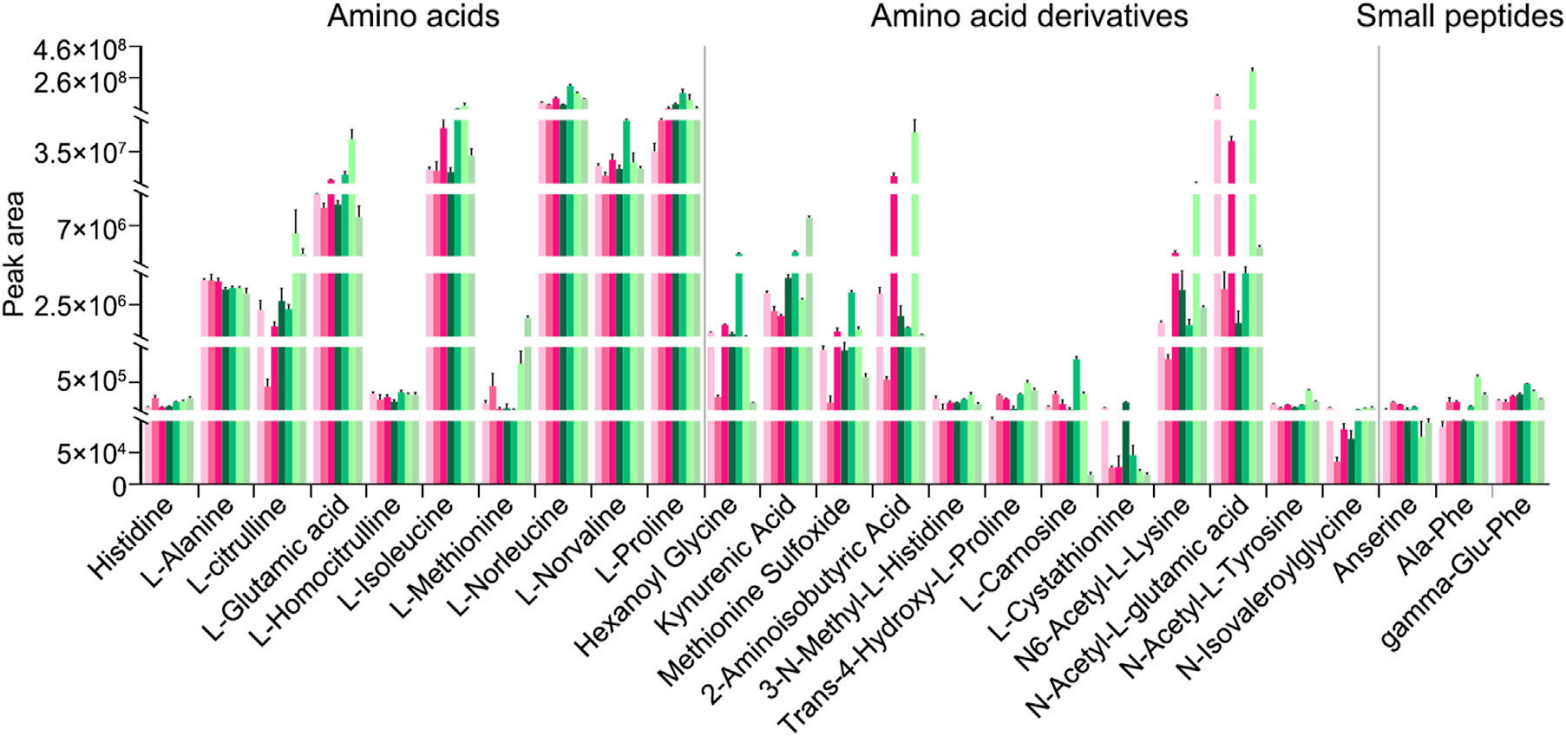
Peak areas of some amino acids and their metabolites that were also absolutely quantified in decoctions of CR, OS, and TS samples; they are also highlighted in bold in Figure 6. Data are presented as mean ± SD, n = 5.

In addition to peak area–based semi-quantitative analysis of metabolites and lipids detected in the decoctions of genuine TCP samples (including CR1, CR2, and CR3) and substituted samples (including OS1, OS2, TS1, and TS2) from different origins, absolute quantitative analyses of 27 amino acids (Figure 6A), 22 amino acid derivatives (Figure 6B), and 18 small peptides (Figure 6C) were carried out. Quantitative results clearly showed that (1) the content of these amino acids, amino acid derivatives, and small peptides in decoctions of 10 g of powdered tortoise shell reached up to 0–69.6 mg, and (2) the content of small peptides was less than that of amino acids and amino acid derivatives by at least an order of magnitude (Figure 6; Supplementary Table S10). Figure 7 shows peak areas of 10 amino acids, 12 amino acid derivatives, and three small peptides that were also quantified and are highlighted in bold in Figure 6. Among them, several low-abundance amino acids and their metabolites, including L-methionine, N-isovaleroylglycine, and anserine, were not well quantified across all tortoise shell decoction samples (Figures 6, 7). Both semi-quantitative and absolute quantitative analyses showed that the content of these amino acids and their metabolites did not differ in decoctions of CR, OS, and TS samples (Figures 6, 7), suggesting the reliability of peak area–based semi-quantitative analysis for screening out differential metabolites and lipids between CR, OS, and TS samples.

## 4 Discussion

*Chinemys reevesii* (Gray) species–sourced traditional Chinese medicine TCP decoction is medicinal and edible. However, what ingredients are contained in this TCP decoction and its substitutes have not yet been comprehensively analyzed. In this study, we integrated LC-MS/MS–based metabolomic and lipidomic profiling to identify water-soluble metabolites and organic-soluble lipids in water decoction samples of genuine TCP and substituted tortoise shell samples from different origins, then screened out differential metabolites and lipids among them. Compared to previous studies focusing on certain classes of TCP chemical components (Wang et al., 2012; Ren et al., 2024), our study for the first time employed high-throughput metabolomic and lipidomic strategies to comprehensively detect hundreds to thousands of metabolites and lipids in TCP decoctions, providing rich chemical information for future exploration of bioactive constituents and optimal utilization.

Additionally, our results revealed remarkable metabolomic and lipidomic differences not only between TCP (CR1–3) and substituted tortoise shell samples (OS1–2 and TS1–2) but also among TCP samples (CR1–3) from different origins. Such findings indicate that the quality of TCP samples collected from different places might vary, and further suggest that potential adulteration with substituted tortoise shell materials may compromise the therapeutic efficacy of TCP preparations. Enrichment analyses revealed that differential metabolites and lipids between CR1–3, OS1–2, and TS1–2 were mainly localized to various metabolism-related pathways. However, N-methyl-4–aminobutyric acid was the only statistically significant (*p* < 0.01) common differential metabolite between CR1–3, OS1–2, and TS1–2. Accordingly, N-methyl-4–aminobutyric acid may be a new indicator for TCP decoction quality evaluation. Furthermore, since the chemical compositions in the decoctions of different substitutes vary, the impact of mixing different substitutes on the efficacy of TCP decoctions may also differ.

To date, studies on the chemical components of TCP and its substitutes are scarce. Our preliminary results demonstrate the feasibility of using metabolomic and lipidomic strategies to evaluate the quality of genuine TCP and substituted tortoise shells. However, this study has several limitations, as follows: (1) the sample size and diversity of TCP and substituted tortoise shells were limited, and the differential metabolites and/or lipids identified among CR, OS, and TS samples may not be generalizable to other substituted samples; (2) the potential effects of rearing conditions and cultivation periods of *Chinemys reevesii* (Gray), *Ocadia sinensis* (Gray), or *Trachemys scripta elegans* (Wied) on the chemical profiles of their respective tortoise shell decoctions have not been validated; and (3) the compositional differences between the carapace and plastron were not investigated.

## 5 Conclusion

In summary, our work for the first time employed LC-MS/MS–based metabolomic and lipidomic analyses to provide a comprehensive overview of chemical constituents in decoctions of genuine TCP and its substitutes. A total of 1,691 metabolites and lipids were detected in decoctions of tortoise shell samples from *Chinemys reevesii* (Gray), *Ocadia sinensis* (Gray), or *Trachemys scripta elegans* (Wied) species. Furthermore, our preliminary results revealed significant compositional differences among *Chinemys reevesii* (Gray) species–sourced TCP samples from different places and between TCP and substituted tortoise shell samples from *Ocadia sinensis* (Gray) or *Trachemys scripta elegans* (Wied) species. N-methyl-4–aminobutyric acid exhibited a statistically significant difference (*p* < 0.01) between TCP and substituted tortoise shell samples, suggesting its potential as an indicator for TCP quality assessment. Further studies are warranted to validate and expand upon these findings, particularly focusing on elucidating the compositional profile and functional components of TCP, to enhance its therapeutic and preventive potential in disease management.

## Data Availability

The original contributions presented in the study are included in the article/Supplementary Material, further inquiries can be directed to the corresponding authors.

## Glossary

TCP: Testudinis Carapax et Plastrum
FC: fold change
KEGG: Kyoto Encyclopedia of Genes and Genomes
MRM: multiple reaction monitoring
MTBE: methyl-tert-butyl ether
PCA: principal component analysis
PCR: Polymerase chain reaction
QC: quality control
FA: fatty acid
GL: glycerolipid
GP: glycerophospholipid
SP: sphingolipid
ST: sterol lipid
DG: diacylglycerol
TG: triacylglycerol
MG: monoglyceride
FFA: free fatty acid
LPC: lysophosphatidylcholine
LPE: lysophosphatidylethanolamine
LPG: lysophosphatidylglycerol
PA: phosphatidic acid
PC: phosphatidylcholine
PE: phosphatidylethanolamine
PG: phosphatidylglycerol
PI: phosphatidylinositol
PS: phosphatidylserine
SM: sphingomyelin
SPH: sphingosine
Cer: ceramide
CAR: carnitine
HexCer: hexosylceramide
BA: bile acid
CE: cholesteryl ester
Cho: cholesterol
Ala: alanine
Gly: glycine
Tyr: tyrosine
Phe: phenylalanine
Asp: aspartic acid
Glu: glutamic acid
Arg: arginine
Ile: isoleucine
Met: methionine
Leu: leucine
Ser: serine
