# Supplementary material for: Metabolomic and lipidomic profiling of traditional Chinese medicine Testudinis Carapax et Plastrum and its substitutes
Source: Front Pharmacol. 2025 Mar 21;16:1549834. doi: 10.3389/fphar.2025.1549834 (PMC11980632; doi:10.3389/fphar.2025.1549834)
Supplement: Supplementary file 2 [file DataSheet1.docx]

Supplementary Material

# Supplementary Material

## Appendix A. Supplementary Data

Table S1. Amino acid–relevant metabolite standards used for quantitative analysis.

Table S2. Detailed information of water-soluble metabolites detected in decoction samples of TCP and substituted tortoise shells.

Table S3. Detailed information of organic-soluble lipids detected in decoction samples of TCP and substituted tortoise shells.

Table S4. Differential analyses of metabolites and lipids detected in decoction samples of CR1 and CR2.

Table S5. Differential analyses of metabolites and lipids detected in decoction samples of CR1 and CR3.

Table S6. Differential analyses of metabolites and lipids detected in decoction samples of CR2 and CR3.

Table S7. Differential analyses of metabolites and lipids detected in decoction samples of CR1 and OS1.

Table S8. Differential analyses of metabolites and lipids detected in decoctions of CR2 and OS2 samples.

Table S9. Differential analyses of metabolites and lipids detected in decoction samples of CR1 and TS1.

Table S10. Differential analyses of metabolites and lipids detected in decoction samples of CR2 and TS2.

Table S11. Absolute quantitative analysis results of some amino acids and their metabolites in decoction samples of CR1–3, OS1–2, and TS1–2.

Figure S1. KEGG pathway enrichment analyses of common differential metabolites and lipids between CR1–3 and OS1–2 samples (A) as well as between CR1–3 and TS1–2 samples (B). The top 20 enriched pathways as ranked by *p*-value are displayed.


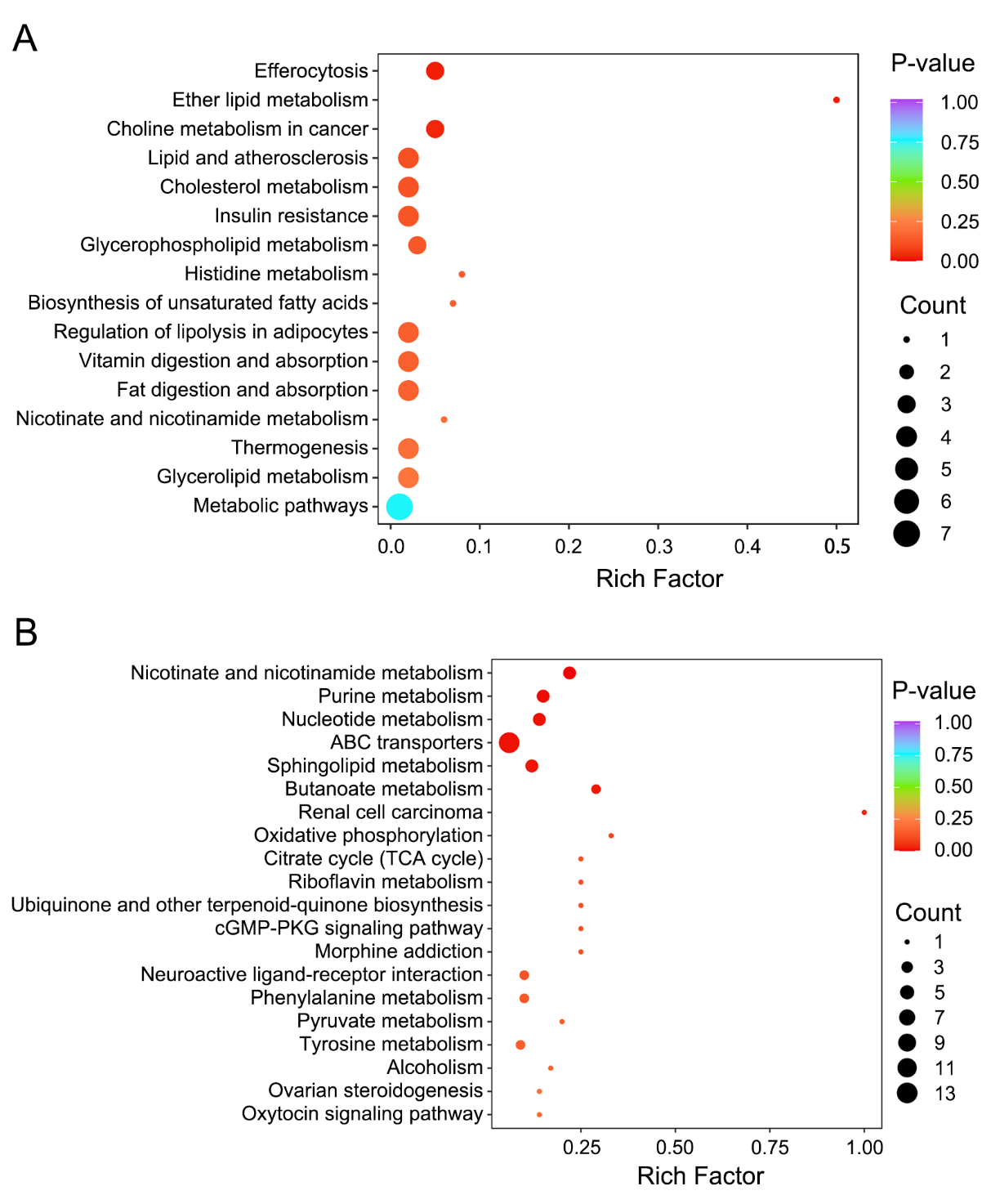


**Figure S1**. KEGG pathway enrichment analyses of common differential metabolites and lipids between CR1–3 and OS1–2 samples (A) as well as between CR1–3 and TS1–2 samples (B). The top 20 enriched pathways as ranked by p-value are displayed.
